# Nanomechanics of the endothelial glycocalyx contribute to Na^+^-induced vascular inflammation

**DOI:** 10.1038/srep46476

**Published:** 2017-04-13

**Authors:** Florian Schierke, Margot J. Wyrwoll, Martin Wisdorf, Leon Niedzielski, Martina Maase, Tobias Ruck, Sven G. Meuth, Kristina Kusche-Vihrog

**Affiliations:** 1 Institute of Physiology II, University of Münster, 48149 Münster, Germany; 2 Department of Neurology, University of Münster, 48149 Münster, Germany

## Abstract

High dietary salt (NaCl) is a known risk factor for cardiovascular pathologies and inflammation. High plasma Na^+^ concentrations (high Na^+^) have been shown to stiffen the endothelial cortex and decrease nitric oxide (NO) release, a hallmark of endothelial dysfunction. Here we report that chronic high Na^+^ damages the endothelial glycocalyx (eGC), induces release of inflammatory cytokines from the endothelium and promotes monocyte adhesion. Single cell force spectroscopy reveals that high Na^+^ enhances vascular adhesion protein-1 (VCAM-1)-dependent adhesion forces between monocytes and endothelial surface, giving rise to increased numbers of adherent monocytes on the endothelial surface. Mineralocorticoid receptor antagonism with spironolactone prevents high Na^+^-induced eGC deterioration, decreases monocyte-endothelium interactions, and restores endothelial function, indicated by increased release of NO. Whereas high Na^+^ decreases NO release, it induces endothelial release of the pro-inflammatory cytokines IL-1ß and TNFα. However, in contrast to chronic salt load (hours), *in vivo* and *in vitro*, an acute salt challenge (minutes) does not impair eGC function. This study identifies the eGC as important mediator of inflammatory processes and might further explain how dietary salt contributes to endothelialitis and cardiovascular pathologies by linking endothelial nanomechanics with vascular inflammation.

High salt (NaCl) intake is increasingly considered a risk factor for inflammatory pathologies and for the development of autoimmune diseases^1^,^2^,^3^,^4^,^5^. In relation to endothelial cells, a functional interface between blood and tissue was recognized to be highly sensitive to salt. Using the atomic force microscope (AFM) tip as a nanosensor it could be demonstrated that, in the presence of aldosterone, a small rise in extracellular Na^+^ concentration by only 7% (from 137 to 147 mM Na^+^) significantly increases the stiffness of the endothelial cortex, an actin-rich layer 50–200 nm beneath the plasma membrane^6^. Under these conditions, release of the vasodilator nitric oxide (NO) from endothelial cells was found to be significantly reduced^6^, indicating salt-induced transition towards endothelial dysfunction. Thus, Na^+^ induces the “stiff endothelial cell syndrome” (SECS)^7^ which in turn promotes inflammatory processes.

The endothelial glycocalyx (eGC), a negatively charged mesh of membranous glycoproteins, proteoglycans, glycosaminoglycans and associated plasma proteins, has recently been recognized as a sodium-sensitive vasoprotective barrier on top of endothelial cells^8^. Moreover, the glycocalyx-polysaccharide surface, which is susceptible to degradation^9^, appears to be a key structure in vascular diseases such as atherosclerosis, stroke, hypertension and chronic kidney disease^10^,^11^. Morphologically, the eGC is linked to proteins of the cortical web beneath the plasma membrane^12^ and thus it is able to transmit biochemical and biomechanical signals from the intravascular compartment to endothelial cells by serving as a critical interface between the blood and vascular wall. In apparent contradiction to its function as a transmitter of signals, the eGC is known to function as a Na^+^ buffering system^13^,^14^. Importantly, the capacity of the eGC to buffer Na^+^ is diminished after exposure to high Na^+^ concentrations. This phenomenon can be explained in part by the fact that Na^+^ strongly interacts with negatively charged side-chains of proteins and proteoglycans within the eGC^15^. Changes in the nanomechanical properties (i.e. stiffness and functional thickness) of the eGC, however, alter its function^16^,^17^. By studying eGC nanomechanics it could be shown that, in the presence of aldosterone, a small increase in the Na^+^ concentration causes collapse of the eGC^13^. A chronic increase in the extracellular Na^+^ concentrations beyond 140 mM ‘neutralized’ the negative charges of the eGC leading to collapse of the structure^13^. Thus, the eGC is highly sensitive to salt, and Na^+^-induced collapse of the eGC results in breakdown of its barrier function. In addition, under high Na^+^ the interaction forces between eGC and red blood cells (RBC) are augmented, which predisposes blood vessels to thrombotic events^18^.

In this study it is hypothesized that the vasoprotective function of the eGC is compromised by high Na^+^ concentrations leading to facilitated leukocyte adhesion and potentiation of vascular inflammation. Since Na^+^ strongly determines the nanomechanical properties of both the eGC and endothelial cortex, a link between endothelial nanomechanics and vascular inflammation is proposed. To test this, a combination of *in vivo* approaches, biochemical studies and specialized AFM techniques was employed.

Here we demonstrate that high extracellular Na^+^ concentrations activate vascular endothelial cells and induce inflammatory processes in the vasculature. In particular, high Na^+^ changes the nanomechanical properties of the eGC and the adhesion forces between monocytes and the endothelial surface. These events are associated with the release of pro-inflammatory cytokines and damage of the eGC. Thus, our data indicate that high Na^+^ impairs the integrity of the lumen-eGC interface and promotes vascular inflammation, providing a promising target for therapeutic intervention.

## Results

### Sodium overload facilitates monocyte adhesion on endothelial cells

Monocyte adhesion is the first step towards vascular inflammation^19^ and it is postulated that high salt has pro-inflammatory properties^3^,^20^. Here we tested in an *in vitro* system whether chronically increased extracellular Na^+^ concentrations trigger inflammatory responses and promote the adhesion of monocytes to vascular endothelium cells.

**Figure 1:**
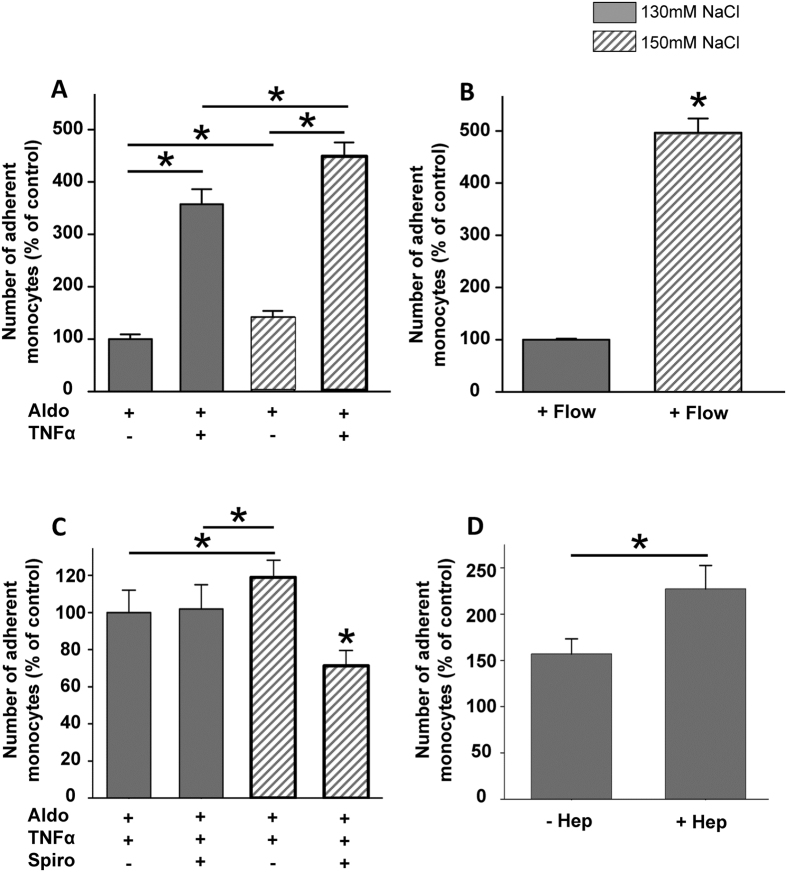
High Na^+^ increases the number of adherent monocytes on endothelial cells. (**A**) In the presence of aldosterone (Aldo) high Na^+^ increases the number of adherent monocytes on an endothelial monolayer. Application of the pro-inflammatory cytokine TNFα enhances the effect. (**B**) Under shear stress (8 dyn/cm^2^) the effect of the Na^+^-induced monocyte adhesion is increased. (**C**) Aldosterone receptor antagonism with spironolactone prevents the Na^+^-induced monocyte adhesion. (**D**) Enzymatic degradation of the endothelial glycocalyx with heparinase (Hep) increases the number of adherent monocyte (N = 4; * indicates p ≤ 0.05).

In the presence of aldosterone (Aldo, 1 nM, 24 h) high extracellular Na^+^ (150 mM, 24 h) applied to endothelial cells (EA.hy926) significantly increased the number of adherent monocytes, compared to low extracellular Na^+^ (130 mM, 24 h) within 4 hours (N = 4; * indicates p = 0.006) (Fig. 1A). Application of TNFα to the cell culture medium had an additive effect on high Na^+^ and resulted in a 4.5 fold increase in adherent monocytes, compared to high Na^+^ alone (N = 3, * indicates p = 0.0008). Under flow (8 dyn/cm^2^) the number of adherent monocytes was increased in general, compared to static conditions. However, incubation in high Na^+^ significantly augmented the adherence of monocytes by about 5 fold, compared to low Na^+^ under flow (N = 3; * indicates p = 0.001) (Fig. 1B). The mineralocorticoid receptor (MR) antagonist spironolactone (Spiro) significantly prevented Na^+^-induced increase in the number of adherent monocytes in the presence of aldosterone (Aldo) and TNFα (N = 3; * indicates p = 0.003) (Fig. 1C), indicating that Spiro confers vasculoprotection under high Na^+^ conditions. Degradation of the eGC with heparinase (Hep) significantly augmented the number of adherent monocytes (Fig. 1D), indicating that the eGC is an important structure governing monocyte adhesion on endothelial cells.

### Sodium overload increases adhesion forces between monocytes and endothelial surface

**Figure 2:**
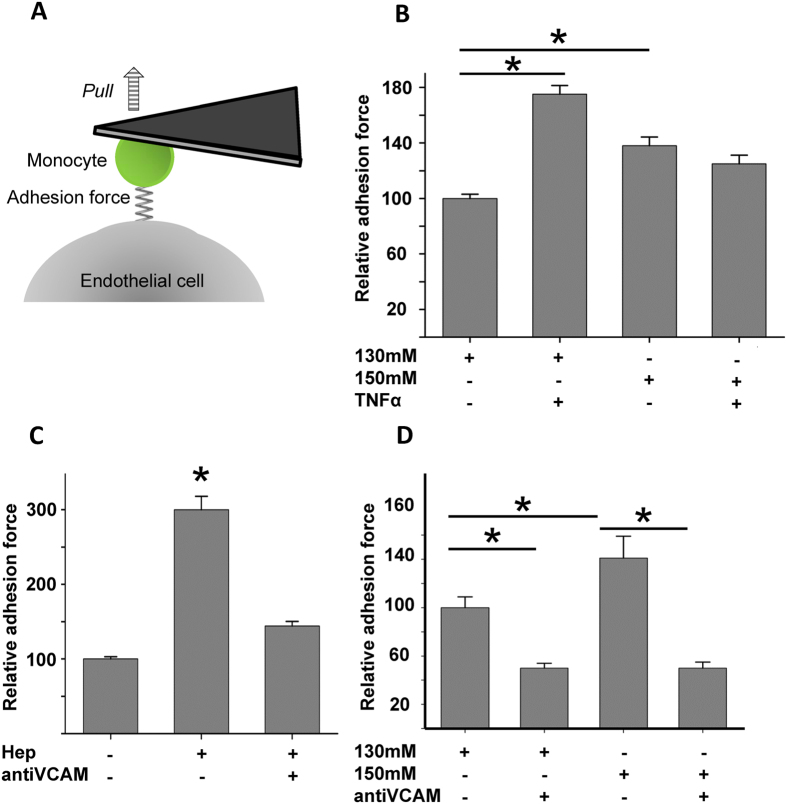
Quantification of the adhesion forces between monocyte and endothelial surface. (**A**) Schematic principle of the CellHesion method. A human monocyte is mounted on a soft cantilever and brought into contact with an endothelial cell. Upon the monocyte touches the surface it is retracted and the unbinding forces are quantified. (**B**) High Na^+^ increased the adhesion forces between monocytes and endothelial surface, compared to low Na^+^ conditions, indicating changed conformation of the eGC and thus stronger binding to surface receptors. Stimulation of the endothelium with TNFα further increased the adhesion forces (N = 6; n = 105–199; * indicates p = < 0.01). (**C**) Enzymatic removal of the eGC with heparinase increased the adhesion forces between monocyte and endothelial surface and could be prevented by application of anti-VCAM antibodies (N = 5; n = 65–93; * indicates p =  < 0.01). (**D**) Blocking of VCAM-1 with specific antibodies prevented the effects of high Na^+^ (N = 6; n = 84–216; * indicates p =  < 0.01).

Adhesion forces between cells were measured using a previously developed specialized AFM technique^18^,^21^,^22^,^23^. This unique method allows the exact quantification of adhesion forces between monocyte and endothelial surfaces and enables the measurement of the mechanical effects of Na^+^ on cell-cell surface interactions *in vitro* (Fig. 2A). The forces necessary to separate both cells were analyzed (nN) and values are given in relative numbers. To test the effects of Na^+^ on adhesion forces, human endothelial cells were incubated for 24 h in low and high Na^+^ (130 mM and 150 mM). As shown in Fig. 2B a significantly greater force (0.22 ± 0.009 nN, +37%) is necessary to separate a human monocyte from the endothelial cell surface with high Na^+^ (150 mM, 24 h), compared to low Na^+^ conditions (130 mM, 24 h, 0.16 ± 0.05 nN) (N = 8, n = 139–185, * indicates p = 0.01). After initiating pro-inflammatory conditions with TNFα the adhesion forces between monocytes and endothelial cells are further augmented (130 mM Na^+^ + TNFα, 0.28 ± 0.02, +75%), indicating an additive effect of Na^+^ and TNFα, likely induced through shedding or damage of eGC. This facilitates the access of monocytes to the endothelial surface (N = 9, n = 190–219, * indicates p = 0.01). Application of TNFα to 150 mM Na^+^ had no additive effect, indicating that the eGC is already shedded by TNFα and cannot be further damaged by Na^+^.

Enzymatic removal of the major components of the eGC (heparan sulfate) with heparinase (Hep)^9^ increases the adhesion forces between monocyte and endothelial surfaces, indicating free access of the monocytes to endothelial surface receptors (Fig. 2C) (0.48 ± 0.03 nN, N = 4, n = 87–95, * indicates p = 0.01). This can be prevented by employing blocking antibodies against VCAM-1 (0.22 ± 0.02, N = 3, n = 103–109, * indicates p = 0.01) (Fig. 2C). Importantly, the effect of high Na^+^ on the adhesion forces between monocytes and the endothelial surface could also be abolished by blocking VCAM (0.24 ± 0.03 nN, N = 3, n = 73–91, * indicates p = 0.01) (Fig. 2D). Since high Na^+^ incubation does not change the protein level of VCAM (Western blot analysis, data not shown), this indicates that under these conditions the accessibility to VCAM at the endothelial surface is increased due to high Na^+^-induced damage to the eGC. In contrast, blocking antibodies against ICAM had no effect on the adhesion forces measured under these conditions (data not shown).

### Sodium overload activates endothelial cells

**Figure 3:**
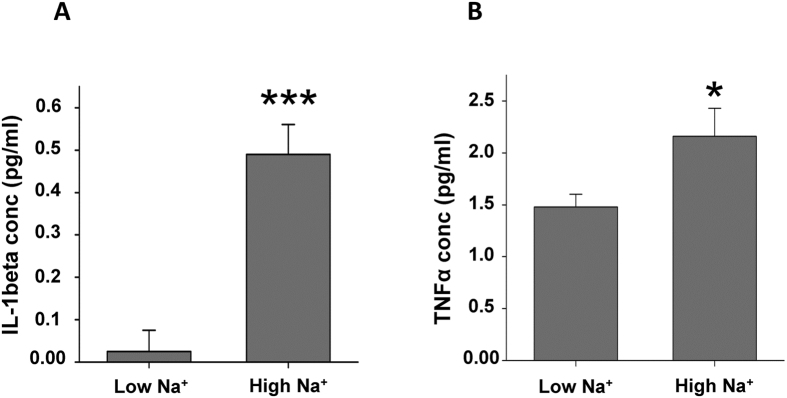
High Na^+^ stimulates the release of proinflammatory cytokines from endothelial cells. Endothelial cells (EA.hy926) were incubated in low and high Na^+^ and the levels of pro-inflammatory cytokines were quantified in the supernatant using the LEGENDplex kit. (**A**) High Na^+^ increases the release of IL1-beta and (**B**) TNFα compared to low Na^+^ (N = 4; * indicates p ≤ 0.05, *** indicates p ≤ 0.001).

Since the adhesion forces between monocytes and endothelial cells are augmented by high Na^+^ we tested whether high Na^+^ induces a shift towards a pro-inflammatory state of the endothelium. Chronic application of high Na^+^ *per se* activates vascular endothelial cells in that the levels of IL-1ß and TNFα are significantly increased after 24 h and 72 h, respectively (Fig. 3, N = 4; * indicates p = 0.01; *** indicates p = 0.001). No effect of high Na^+^ was found on the secretion of IL-6 and MCP-1.

### Chronic sodium overload alters the eGC

**Figure 4:**
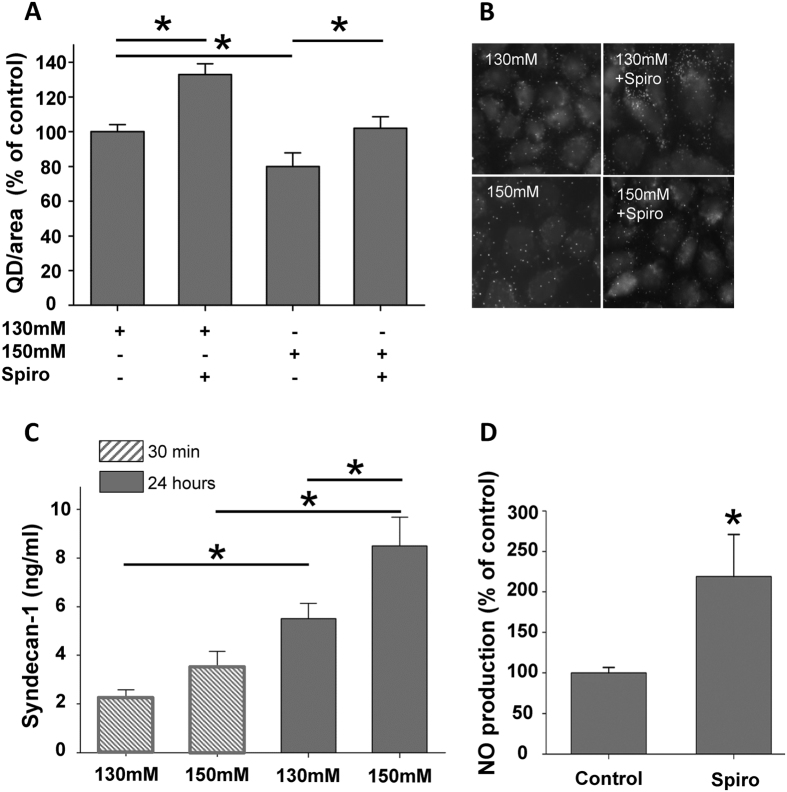
Chronic (24 hours), but not acute (30 min) salt challenge damages the eGC. (**A**) Endothelial cells (EA.hy926) were incubated in low and high Na^+^ for 24 h and heparan sulfates (HS) were stained and quantified with a specific anti-HS antibody and secondary Quantum Dot (QD) antibody. High Na^+^ decreased HS, compared to low Na^+^, application of spironoloctone (spiro) prevented the high Na^+^ effect (N = 3; n = 60, * indicates p ≤ 0.05). (**B**) Representative images of QD-mediated stainings of HS in endothelial cells. (**C**) Levels of syndecan-1 were detected in the supernatant of endothelial cells after stimulation with Na^+^ using an ELISA. Shedding of the eGC (high syndecan-1 levels) was observed only in chronically (24 h) treated endothelial cells but not with acute (30 min) incubation in high Na^+^ (N = 6; * indicates p ≤ 0.05). (**D**) The NO release of endothelial cells was measured as marker for endothelial function. Spironolactone increased the endothelial production of NO, indicating improvement of vascular function (N = 4; * indicates p ≤ 0.05).

**Figure 5:**
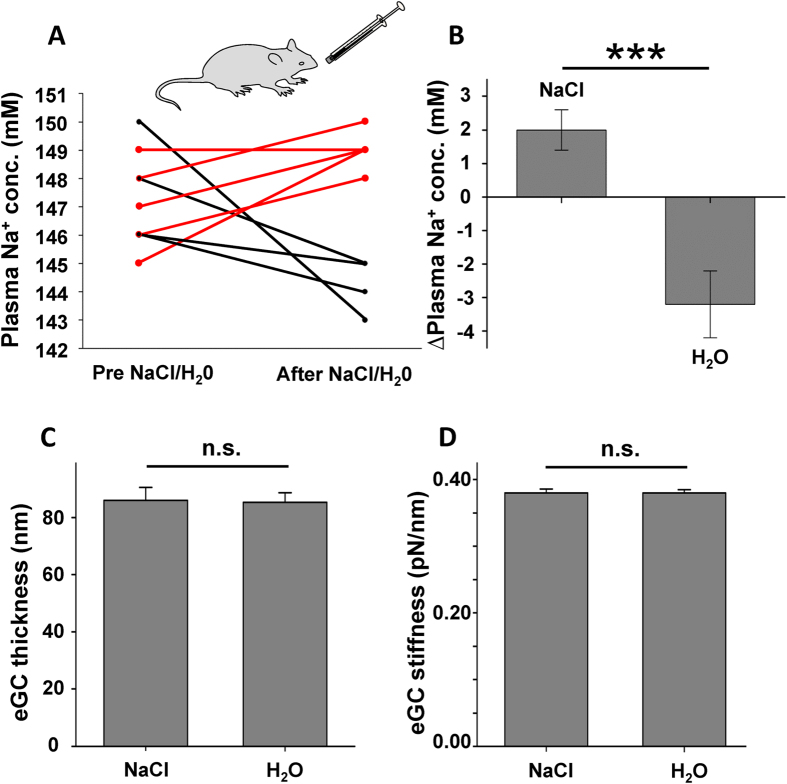
Acute oral salt challenge increases the plasma Na + concentration. (**A**) Plasma Na^+^ concentrations were measured after oral application of salt/glucose solution or water with a stomach tube in the blood plasma of wild-type mice. 30 minutes after the treatment the salt/glucose uptake resulted in an increased plasma Na^+^ concentration while water intake decreased the plasma Na^+^ concentration. (**B**) Differences between the plasma Na^+^ concentration before and after salt/glucose or water intake (N = 5, *** indicates p ≤ 0.001). No acute effects of salt/glucose intake on the thickness **(C)** and stiffness **(D)** of the endothelial glycocalyx could be observed in *ex vivo* endothelial cells of aorta preparations measured with the AFM (N = 5, n = 81–90).

The stability of the eGC strongly depends on the sodium concentration, hence a small rise in extracellular Na^+^ concentration (+2–5 mM) leads to a collapse of the structure^13^. In the present study we could show using immunofluorescence microscopy and a specific anti-heparane sulfate (HS) antibody that 24 h sodium overload reduces the HS content (−20%; N = 4; n = 101–122; * indicates p = 0.02), a major component of the eGC, consistent with collapse or shedding of the eGC^9^,^13^ (Fig. 4A). Aldosterone receptor antagonism with spironolactone prevents loss of HS (+22%; N = 4; n = 122–136; * indicates p = 0.02) (Fig. 4A). Figure 4B shows representative images of HS immunostainings of endothelial cells under the following conditions (i) 130 mM Na^+^, (ii) 130 mM Na^+^ + spironolactone (100 nM), (iii) 150 mM Na^+^ and (iv) 150 mM Na^+^ + spironolactone (100 nM). As a marker for eGC damage, syndecan-1 was chosen since it represents an important core protein of the eGC. Incubation of endothelial cells in low and high Na^+^ revealed that chronic application (24 hours) leads to markedly increased levels of syndecan-1, consistent with shedding of the eGC due to Na^+^ overload (Fig. 4C) (N = 5; * indicates p = 0.02). Acute Na^+^ excess (30 min), however, did not impair the structure of the eGC (Figs 4C and 5C,D). To test the functional consequences of MR antagonism, NO production from endothelial cells was quantified. Indeed, spironolactone treatment significantly increased NO production by 120%, compared to control conditions (N = 6; * indicates p = 0.02) (Fig. 4C), indicating improvement of endothelial function.

### Acute oral salt challenge increases plasma Na^+^ concentration *in vivo*

Application of hypertonic salt/glucose solution (1.6 g NaCl/3 g glucose) via a stomach tube increased the plasma sodium concentration of wild type mice by 2 mM, from 147.0 ± 0.71 mM to 149.0 ± 0.31 mM (N = 5; * indicates p = 0.03), whereas the same amount of tap water lowered the plasma sodium concentration by 3 mM, from 147.6 ± 0.74 mM to 144.4 ± 0.4 mM (N = 5; * indicates p = 0.005). This is in agreement with a clinical study^24^. Changes of plasma sodium concentration for individual mice are depicted in Fig. 5A. The mean values of the measurements are shown in Fig. 5B.

We developed an AFM-based nanoindentation protocol which enables us to quantify the functional thickness and individual stiffness of the eGC^13^. By analysing the force-distance curves of single endothelial cells it was shown that chronic exposure to high ambient Na^+^ (5 days) changes the nanomechanical properties of the eGC in that it stiffens and flattens^13^. In the present study the mechanical stiffness of *ex vivo* endothelial cells derived from aortae dissected 30 min after oral application of salt/glucose was quantified as previously described^25^. Whereas oral Na^+^ challenge increased the plasma Na^+^ concentration in mice by about 2 mM (Fig. 5A), it did not affect the nanomechanical properties (thickness and stiffness) of the eGC *ex vivo* (Fig. 5C,D). Also, acute application of a high Na^+^ concentration (150 mM, 30 min) *in vitro* did not change the eGC structure measured using AFM (data not shown), indicating a preserved mechanism which makes the eGC resistant to (naturally occurring) changes in plasma Na^+^ concentration (e.g. due to a salty meal). In contrast, the mechanical properties of the endothelial cortex are known to be sensitive to increasing Na^+^ concentrations (from 135 mM to 150 mM) within minutes^26^.

## Discussion

Since mechanical stiffening of endothelial cells strongly contributes to impaired NO release^27^, we propose that Na^+^-induced modifications of the physical properties of the outer layer of endothelial cells (eGC and cortex) are early events of endothelial dysfunction which may culminate in chronic inflammatory diseases.

Here, we report that high physiological Na^+^ concentrations, as present in the plasma after oral salt challenge, e.g. a salty meal^24^, initiate a MR-mediated mechanistic cascade leading to endothelial stiffening, dysfunction and vascular inflammation. The AFM was employed as a nanosensor to quantify the adhesion forces and the mechanical stiffness of living endothelial cells *ex vivo* and *in vitro*. We found that (i) high Na^+^ strengthens the adhesion forces between monocytes and the endothelial surface *in vitro*, (ii) chronic (24–72 hours), but not acute (30 min) salt excess damages the eGC *ex vivo* and (iii) high Na^+^ induces the release of pro-inflammatory cytokines from endothelial cells. Aldosterone receptor antagonism with spironolactone prevents Na^+^-induced endothelial dysfunction. Our results underscore that high Na^+^ disturbs eGC barrier function and predisposes the endothelium to inflammation.

Recently, the eGC was recognized as an important structure during leukocyte recruitment^28^,^29^. It is postulated that it has both pro-adhesive and anti-adhesive functions and thus plays a crucial role during inflammatory processes^28^,^30^. Due to its position on the surface of endothelial cells the eGC serves as a ‘firewall’ by mediating flow-induced shear stress and regulation of leukocyte-endothelium interactions. Since the eGC can reach a height of about 0.5–1 μm, leukocytes are ‘tip-toeing’ with their cytoskeletal protrusions on the eGC and can barely reach the adhesion molecules at the endothelial surface - unless the barrier is compromised. In the present study the importance of the eGC was demonstrated by the observation that enzymatic removal of the eGC enhances adhesion forces between monocytes and the endothelial surface in a VCAM-dependent (but not ICAM-dependent) manner. This indicates that a ‘healthy’ eGC prevents the formation of bonds between binding partners on monocytes and endothelial surfaces. In addition, we report that monocyte adhesion is facilitated after chronic treatment of vascular endothelial cells with high Na^+^, which can be prevented by using VCAM antibodies. The underlying explanation appears to be Na^+^-induced conformational changes and damage of the eGC. Since the protein level of VCAM was not further increased with high Na^+^ in the presence of TNFα, we conclude that high Na^+^ creates a condition whereby the accessibility to the endothelial surface is facilitated via damage to the eGC barrier. This, in turn, strengthens the adhesion forces between monocytes and the endothelial surface. Our data are in agreement with previous studies showing that high Na^+^ promotes the adhesion and pro-atherogenic effects of leukocytes^31^.

Physical detachment of two cells (e.g. the detachment of a leukocyte from an endothelial cell) involves a series of single rupture events. Each event represents the unbinding of one or more adhesive ligand-receptor bonds. The magnitude of these unbinding steps lies in general between 40 and 100 pN^18^,^32^. Since the unbinding forces (maximal adhesion force) between monocyte and endothelial surfaces measured in the present study were in the range of 0.2–0.4 nN, we postulate that one monocyte binds to many binding sites.

Thus, the Na^+^-dependent increase in adhesion forces between monocytes and the endothelial surface found in the present study might be explained by more favorable interactions between molecular binding partners conferred by high Na^+^-induced changes in the eGC nanomechanics. Notably, we found that the treatment of cells with heparinase to enzymatically remove the eGC results in higher unbinding forces than application of high Na^+^ (0.54 ± 0.04 nN vs. 0.22 ± 0.009 nN). This indicates two different underlying mechanisms. As mentioned earlier, heparinase removes a major component of the eGC which improves the access of monocyte receptors to adhesion molecules on the endothelial surface and results in strong (un)binding forces. A second Na^+^-dependent mechanism is postulated in which monocytes bind with their ligand L-selectin to heparan sulfate proteoglycans directly within the endothelial glycocalyx^29^,^33^,^34^. This results in lower binding forces and can be seen as the first step of the monocyte adhesion cascade. It is concluded that heparinase induces shedding of the eGC while high extracellular Na^+^ causes a conformational change. This is in agreement with previous reports showing that Na^+^ treatment leads to a stiff and flat eGC^13^. In the setting of inflammation a similar observation was made. Circulating leukocytes are captured by adhesive tethering of surface L-selectins to opposing P- and E-selectins on the endothelial surface (eGC), followed by rolling and adhesion of the leukocyte to the endothelium. These processes require the activation of both the leukocytes and the endothelium, including the expression of specific adhesion molecules^35^,^36^.

During inflammation endothelial cells are activated by cytokines which induces the expression of adhesion molecules^35^. In the present study, however, we could demonstrate that high Na^+^ concentrations *per se* activate endothelial cells by direct induction of pro-inflammatory IL-1-β and TNF-α secretion. The combination of a compromised eGC barrier and endothelial activation after salt excess is most likely responsible for the increased attraction of monocytes to the endothelial surface.

As previously demonstrated, increased plasma Na^+^ has high pro-inflammatory potential through both enhancing proinflammatory responses and impairing regulatory mechanisms^1^,^37^,^38^. It turned out that the vascular endothelium is sensitive to a small rise in Na^+^ concentrations, such that a defined range of extracellular Na^+^ concentrations tightly links the mechanical properties to NO production^6^. In this context, the endothelial Na^+^ channel (EnNaC) was identified as an important regulatory mediator of endothelial stiffness^39^,^40^. In particular, an acute rise in plasma Na^+^ by 2–3 mM stiffens the endothelial cortex^26^, but does not influence the structure/function of the eGC (this study). Neither changes of stiffness/height of the eGC, used as structural markers, nor shedding of the eGC, a functional marker, were found after 30 min of oral salt challenge *in vivo*. This indicates that the eGC is resistant against short-term Na^+^ load, while the endothelial cortex immediately reacts to changes in extracellular Na^+^. Since regular food intake is coupled to slightly increased plasma Na^+^ concentrations^24^, this stability of the eGC against acutely increased Na^+^ is of high physiological relevance. In contrast, chronically increased plasma Na^+^ concentrations, due to, for example, constantly increased salt load or dehydration over days, might lead to pathophysiological changes in eGC structure, paralleled by impaired vascular function.

Recently, the MR antagonist spironolactone was shown to improve endothelial function by softening the endothelial cortex^41^ and preventing the eGC from damage by high Na^+^^13^. In the present study, MR antagonism prevented the pro-adhesive effects of high Na^+^ on monocytes. This might be related to the finding that spironolactone prevents high Na^+^-induced damage to the eGC^13^. Furthermore, we provide evidence that spironolactone facilitates the endothelial release of NO and thus preserves vascular function.

These findings again indicate that aldosterone sensitizes endothelial cells for Na^+^. Recently, we could show that aldosterone directly acts on endothelial cells and induces the membrane insertion of EnNaC which stiffens the endothelial cortex (for review see ref. 42). Thus we propose the following sequence of events: In the presence of aldosterone, high Na^+^ triggers the membrane insertion of EnNaC leading to increased mechanical stiffness of the endothelial cortex and reduced release of NO. In parallel, aldosterone/Na^+^ damages the eGC. This MR-mediated mechanism chronically leads to endothelial dysfunction and vascular inflammation.

In summary, our findings suggest that high Na^+^ conditions, besides contributing to the development of endothelial dysfunction, activate vascular endothelial cells and create pro-inflammatory conditions (endothelialitis). In this context it can be stated that endothelial nanomechanics contribute significantly to proper endothelial function. Hence, high Na^+^-induced changes in the nanomechanical properties of the eGC and cortex contribute to the development of inflammatory processes, making them both a predictor and therapeutic target for vascular pathologies.

## Methods

### Animals and diets

All procedures were approved by a governmental committee on animal welfare (Landesamt für Natur, Umwelt und Verbraucherschutz Nordrhein-Westfalen), performed according to international animal protection guidelines and all efforts were made to minimize suffering.

Ten to twenty weeks old male C57BL6/6 J WT mice with 20–25 g body weight were used for the experiments. They received a standard chow and free access to plain tap water up to 24 h prior to the experiment. For the experiments mice were carefully gavaged using a stomach tube either with about 200 μl hypertonic saline (1.6 g NaCl plus 3 g glucose/100 ml tap water) or tap water. About 100 μl blood was collected from the animals under short-time anaesthesia via punctuation of the retrobulbar venous plexus before and after the oral salt/water challenge within a time frame of two weeks. Plasma sodium concentrations were measured by using an electrolyte analyzer (ABL 800; Radiometer, Copenhagen).

### Aorta preparation and culture

Animals were anesthetized by isoflurane inhalation, followed by terminal interruption of the circulation. After opening the thorax, the ascending aorta was fixed with fine forceps and separated from the heart. The thoracic aorta was then dissected from the aortic arch to the diaphragm. Cleaning of connective tissue under microscopic visualization and further preparations were conducted as recently described^25^,^39^,^43^. For AFM measurements e*x vivo* aorta preparations were glued on glass coverslips using Cell-Tak^TM^ (BD Biosciences) and cultured at 37 °C and 5% CO_2_ in minimal essential medium (MEM, Invitrogen) containing 1% MEM vitamins (Biochrom), penicillin G (10,000 U/mL), streptomycin (10,000 μg/mL) and 1% MEM non-essential amino acids (MEM NEAA, Gibco).

### Endothelial cell culture and isolation of human monocytes

Human endothelial EA.hy 926 cells (kindly provided by Cora-Jean S. Edgell, University of North Carolina, Chapel Hill, NC, USA) were grown in culture as described elsewhere^44^. Briefly, endothelial cells were cultured in T25 culture flasks using DMEM medium (Invitrogen Corp., Karlsruhe, Germany) supplemented with NaHCO_3_, penicillin G, streptomycin (Biochrom AG, Berlin, Germany) and fetal bovine serum (FCS) (PAA Clone, Coelbe, Germany). For experiments, cells were seeded on thin glass coverslips (Ø = 15 mm) and used after reaching confluence (48–72 h). Endothelial cells were incubated in low sodium (130 mM) or high sodium (150 mM) 24 h prior to the experiment. Differences in osmolarity between 130 mM and 150 mM NaCl solutions were substituted with mannitol. To prevent a loss of water and to maintain constant sodium concentrations in the medium, culture dishes were placed in a water-filled chamber inside a standard incubator. Chemicals were added to the medium as appropriate.

GM7373 cells (DSMZ GmbH, Braunschweig, Germany), an immortalized endothelial cell line from bovine aorta^45^, were cultured as described elsewhere^46^. Briefly, cells were kept at 37 °C, 5% CO_2_ and 95% humidity in T25 cell culture flasks using minimal essential medium (MEM; GE Healthcare/PAA, Pasching, Austria) supplemented with 20% bovine fetal serum (BFS; PAA, Pasching, Austria), 1% MEM vitamins (Gibco/Thermofisher Scientific, Massachusetts, USA), 1% penicillin and streptomycin (10000 U/ml or 10000 μg/ml respectively; Biochrom, Berlin, Germany) and 1% MEM non-essential amino-acids (Gibco/Thermofisher Scientific, Massachusetts, USA). For experiments cells between passages 5 and 18 were used.

Human monocytes were isolated using the pluriSelect monocyte isolation kit (anti-hu CD14, Hiss diagnostics, Freiburg, Germany) via the principle of positive selection from blood samples of healthy donors according to the manufacturer’s protocol.

### Atomic Force Microscopy (AFM) measurements

Mechanical stiffness and thickness of the endothelial glycocalyx was determined using an AFM nano-indentation technique^47^,^48^. Stiffness measurements of living endothelial cells *ex vivo* were conducted with a scanning probe microscope (MultiMode^®^ SPM, Bruker, Karlsruhe, Germany) with a feedback-controlled heating device (Bruker) During the measurements, artery preparations were bathed in HEPES-buffered solution (standard composition in mM: 135 NaCl, 5 KCl, 1 MgCl_2_, 1 CaCl_2_, 10 HEPES (N-2-hydroxyethylpiperazine-N′-2-ethanesulfonicacid), pH 7.4). AFM nanoindentation measurements were performed using soft cantilevers (spring constant: <20 pN/nm; Novascan, Ames, IA, USA) with a polystyrene sphere as the tip (diameter: 10 μm). A maximal loading force of 2 nN was applied. Obtained AFM data were collected with NanoScope softwares 5.31 and V8.10 (Bruker). Stiffness and thickness values were calculated from force-distance curves using the Protein Unfolding and Nano-Indentation Analysis Software PUNIAS 3D version 1.0 release 2.2 (http://punias.voila.net).

Adhesion measurements were performed by using a CellHesion 200 instrument (JPK, Berlin, Germany) equipped with a Petri dish heater for maintaining 37 °C as recently described^18^. Arrow TL-1 tipless cantilevers (NanoAndMore GmbH, Wetzlar, Germany) were incubated prior to all experiments for 20 min in Cell-Tak (BD Biosciences, Bedford, Massachusetts, USA) to attach an isolated human monocyte to the tipless cantilever (Fig. 2A). To quantify the adhesion forces, the monocyte was brought into contact with the endothelial surface for 1 sec with a constant loading force of 1 nN. Force-distance curves were obtained by probing EA.hy926 cells with a monocyte-carrying cantilever. In average, two force-distance curves were performed per one individual cell. Maximal adhesion forces between monocyte and endothelial surface, represented by the rupture of the bonds between both surfaces, are analyzed (given as relative values) using the JPK Data Processing (software version 4.2.50). During the whole procedure the endothelial cell layer was always covered by HEPES buffered saline.

### Quantum dot (QD)-based immunofluorescence of heparan sulfate at cell surface

As previously described human endothelial cells (EAhy.926) were fixed in a non-permeabilizing manner with 0.1% glutaraldehyde and stained as described elsewhere^13^,^49^ Briefly, after blocking the fixed cells with 10% normal goat serum, the primary heparan sulfate antibody (Seikagaku Corporation, Tokyo, Japan; 1:1000) was applied for 1 h at room temperature. After washing, a secondary QD-labeled antibody (Qdot 655 goat anti-rabbit IgG, Invitrogen; 1:800) was used for staining. Negative controls were established by incubating cells solely with the secondary antibody. Staining was verified by epifluorescence microscopy (microscope: Leica DMI 6000B, Leica Microsystems; camera: CoolSNAPHQ, Photometrics). The QD-based immunofluorescence was quantified by counting QD/area of cell surface using ImageJ software (National Institutes of Health, USA). Images were taken in 3 different sections of the endothelial monolayer and all images were analyzed simultaneously in order to account for any variations in cell height. QD background levels (QD detected in negative controls) were subtracted from the results.

### Monocyte adhesion assay

EA.hy926 cells were incubated in low or high Na^+^ medium for 24 hours containing 1 nM aldosterone. Isolated human monocytes were fluorescently labelled with an Alexa Flour 488 anti-human CD14 antibody (Biolegend, San Diego, USA) and added to the EA.hy926 monolayer for 4 hours. To remove non-adherent monocytes cells were washed carefully four times with phosphate buffered saline (PBS, in mM: 140 NaCl, 2 KCl, 4 Na_2_HPO_4_, 1 KH_2_PO_4_, pH 7.4) following a standardized protocol. Cells and adherent monocytes were fixed with 4% paraformaldehyde (PFA) and subjected to fluorescence microscopy for further analysis.

For quantification of monocyte adhesion on endothelial cells under flow the IBIDI pump system was employed. Fluorescently labelled EA.hy926 cells were seeded onto μ-slides (IBIDI, Martinsried, Germany) and unidirectional shear stress (8 dyne/cm^2^) was applied for 24 h hours. Afterwards, cells were fixed as described above and adherent monocytes were quantified using a fluorescence microscope (microscope: Leica DMI 6000B, Leica Microsystems; camera: CoolSNAPHQ, Photometrics).

### Quantification of glycocalyx components

Syndecan-1 levels of EA.hy926 supernatants were determined directly using a sandwich enzyme immunoassay with an antibody specific to human Syndecan-1 (Diaclone, Besancon Cedex, France) according to the manufacturer’s instructions. Cells were chronically incubated in medium containing 130 mM or 150 mM Na^+^ 24 h prior to the experiments. All measurements were performed in duplicates.

Mouse plasma levels of Syn-1 were measured with a sandwich ELISA kit specific for mouse Syndecan-1 (Cell Sciences^®^, Canton, MA, USA). Blood samples before and after oral Na^+^/water challenge were used in duplicate.

### Cytokine quantification

To test the hypothesis that high Na^+^ activates endothelial cells and thus stimulates the release of proinflammatory cytokines, EA.hy926 cells were incubated in medium containing (i) 130 mM + mannitol and (ii) 150 mM Na^+^ for both 24 h and 72 h in a humid chamber. The concentration of the proinflammatory cytokines IL-1β, IL-6, TNF-α and CCL-2 were measured in the in cell culture supernatants employing the LEGENDplex Kit according to the manufacturer’s instructions (Biolegend, San Diego, USA) as described before^50^.

### Chemiluminescent detection of the NO concentration in the cell culture supernatant

To quantify NO production of endothelial cells nitrates and nitrites, the stable breakdown products of NO, can be detected with the NO-analyzer CLD 88 (Eco Medics, Duernten, Switzerland) in the cell culture supernatant. In this method, vanadium(III)-chloride induces a reduction of all nitrates and nitrites to NO at 95 °C. After contact of the NO with ozone (produced by a generator inside the setup) a photon is emitted, which can be detected as chemiluminescence^51^. Experiments were performed with GM7373 cells kept on a shaker (33 rounds per minute, angle 11°) inside the incubator (37 °C, 5% CO_2_ and 95% humidity) to stimulate NO synthesis and release (control and spironolactone treated). 50 μl of the supernatant were injected into the vanadium(III)-chloride (Sigma-Aldrich, München, Germany) filled reaction chamber of the CLD 88 setup, whereas triplicates of all samples were measured. For analysis, from every resulting peak the area under the curve was determined with the eDAQ Chart software (https://www.edaq.com/software-downloads). Subsequently, the slope of the standard curve was calculated in order to quantify the amount of NO from every injected sample. The blank value (solely medium) was subtracted from all values and the mean of the triplicates was calculated. Results are presented as percentage of control.

### Statistics

Significance of difference in the statistics was determined using one-way ANOVA and two-sample Student’s t-test in case of a normally distributed population or using Kruskal Wallis ANOVA and Mann-Whitney test for populations that did not follow a normal distribution (*p < 0.05). All depicted data were calculated as median (±standard deviation) or as mean (±SEM), depending on normal distribution.

## Additional Information

**How to cite this article**: Schierke, F. *et al*. Nanomechanics of the endothelial glycocalyx contribute to Na^+^-induced vascular inflammation. *Sci. Rep.* **7**, 46476; doi: 10.1038/srep46476 (2017).

**Publisher's note:** Springer Nature remains neutral with regard to jurisdictional claims in published maps and institutional affiliations.
